# A Brain-Inspired Theory of Mind Spiking Neural Network for Reducing Safety Risks of Other Agents

**DOI:** 10.3389/fnins.2022.753900

**Published:** 2022-04-14

**Authors:** Zhuoya Zhao, Enmeng Lu, Feifei Zhao, Yi Zeng, Yuxuan Zhao

**Affiliations:** ^1^Research Center for Brain-Inspired Intelligence, Institute of Automation, Chinese Academy of Sciences, Beijing, China; ^2^School of Future Technology, University of Chinese Academy of Sciences, Beijing, China; ^3^School of Artificial Intelligence, University of Chinese Academy of Sciences, Beijing, China; ^4^National Laboratory of Pattern Recognition, Institute of Automation, Chinese Academy of Sciences, Beijing, China; ^5^Center for Excellence in Brain Science and Intelligence Technology, Chinese Academy of Sciences, Shanghai, China

**Keywords:** brain-inspired model, safety risks, SNNs, R-STDP, theory of mind

## Abstract

Artificial Intelligence (AI) systems are increasingly applied to complex tasks that involve interaction with multiple agents. Such interaction-based systems can lead to safety risks. Due to limited perception and prior knowledge, agents acting in the real world may unconsciously hold false beliefs and strategies about their environment, leading to safety risks in their future decisions. For humans, we can usually rely on the high-level theory of mind (ToM) capability to perceive the mental states of others, identify risk-inducing errors, and offer our timely help to keep others away from dangerous situations. Inspired by the biological information processing mechanism of ToM, we propose a brain-inspired theory of mind spiking neural network (ToM-SNN) model to enable agents to perceive such risk-inducing errors inside others' mental states and make decisions to help others when necessary. The ToM-SNN model incorporates the multiple brain areas coordination mechanisms and biologically realistic spiking neural networks (SNNs) trained with Reward-modulated Spike-Timing-Dependent Plasticity (R-STDP). To verify the effectiveness of the ToM-SNN model, we conducted various experiments in the gridworld environments with random agents' starting positions and random blocking walls. Experimental results demonstrate that the agent with the ToM-SNN model selects rescue behavior to help others avoid safety risks based on self-experience and prior knowledge. To the best of our knowledge, this study provides a new perspective to explore how agents help others avoid potential risks based on bio-inspired ToM mechanisms and may contribute more inspiration toward better research on safety risks.

## 1. Introduction

With the vigorous advancement of AI, applications such as self-driving cars and service robots may widely enter society in the future, but avoiding risks during interaction has not been solved yet. As humans, we will help others when they may run into danger. Understanding and inferring others' actions contribute to avoiding others suffering from safety risks. For humans, the ability to make inferences about beliefs and motivations is called the theory of mind (ToM) (Sebastian et al., 2012; Dennis et al., 2013). ToM is also considered as the ability to understand of the difference between your own beliefs and that of others (Shamay-Tsoory et al., 2007). ToM seems to depend on a group of brain areas, which mainly includes the temporo-parietal junction (TPJ), part of the prefrontal cortex (PFC), the anterior cingulate cortex (ACC), and the inferior frontal gyrus (IFG). The ACC evaluates others' state values (Abu-Akel and Shamay-Tsoory, 2011). The IFG is a critical area for the inhibition process: self-perspective inhibition (Hartwright et al., 2012; Hartwright et al., 2015). The TPJ and the PFC are two important areas for ToM which are related to perspective taking and represent others' traits (Koster-Hale and Saxe, 2013), respectively. Motivated by this, this article aims to develop a brain-inspired ToM model to infer others' false beliefs and policies to reduce others' safety risks.

The prediction sources are fundamental and crucial to ToM (Koster-Hale and Saxe, 2013). One source of predictions about a person's beliefs and desires is the action (Patel et al., 2012; Zalla and Korman, 2018). The individuals expect other people to be self-consistent and coherent. Besides, self-experience is mentioned as self-projection (Buckner and Carroll, 2007; Patel et al., 2012) or using memories to understand others. Therefore, we combined prior knowledge of others and self-experience to perceive others' states and predict their behaviors.

Taking inspiration from the multi-brain areas cooperation and neural plasticity mechanisms of ToM, this article proposes a biologically realistic ToM spiking neural network model, namely, a brain-inspired ToM spiking neural network (ToM-SNN) model. We designed the structure of our model with neuroanatomical and neurochemical bases of ToM. The ToM-SNN model consists of four parts: the perspective taking module (TPJ and IFG), the policy inference module (vmPFC), the action prediction module (dlPFC), and the state evaluation module (ACC). The output of each submodule is interpretable. We embedded the model into an agent and focused on the problem of how to use the ToM-SNN model to reduce safety risks based on self-experience (Zeng et al., 2020) as well as prior knowledge of others acquired through direct interaction (Koster-Hale and Saxe, 2013).

The innovative aspects of this study are as follows.

(1) Inspired by the ToM information processing mechanism in the brain, we proposed multi-brain areas coordinated SNNs model, including the TPJ, the PFC, the ACC, and the IFG. We adopted STDP and Reward-modulated Spike-Timing-Dependent Plasticity (R-STDP) training different modules based on their functions. Therefore, our training methods are more biologically plausible than artificial neural network training methods, such as backpropagation.

(2) Our experimental results show that the ToM-SNN model can distinguish self-and-other perspectives, infer others' policy characteristics, predict others' actions, and evaluate safety status based on self-experience and prior knowledge of others. The agent with the ToM-SNN model can help others avoid safety risks timely. Compared with experiments without the ToM-SNN model, agents behave more safely in the experiments with the ToM-SNN model. In addition, the model will behave differently for agents with different policies to help others as much as possible while minimizing their losses.

(3)To the best of our knowledge, this is the first study to investigate the application of the biological realistic ToM-SNN model on safety risks.

The rest of this article is organized as follows. Section 2 gives a brief overview of the related work of safety risks and the ToM computational model. Section 3 is concerned with the methodology proposed in this article for this study. Section 4 introduces the exact experiment procedure and analyses the results of experiments. Some discussions and conclusions are in section 5.

## 2. Related Studies

### 2.1. Safety Risks

Artificial Intelligence Safety can be broadly defined as the endeavor to ensure that AI is deployed in ways that do not harm humanity. With the rapid development of AI, many AI technologies are gradually applied to social life in recent years. Compared with the wide application of perceptual AI, cognitive AI in real life is less common. The reason is that the actual environment is complex and changeable, increasing the model robustness requirement. So before these technologies are widely used, it is necessary to explore the safety risks of these technologies.

To avoid the application risk of AI technology in the future, many researchers carried out a series of research on AI Safety. Amodei et al. (2016) put forward the problems that need to be considered in AI Safety: avoiding negative side effects, avoiding reward hacking, scalable oversight, safe exploration, and robustness to distributional shift. Similarly, Leike et al. (2017) divided AI Safety problems into two categories: value alignment and robustness. Value alignment mainly refers to four problems caused by the inconsistency between goals of human and artificial agents: safe interruptibility, avoiding side effects, absent supervisor, and reward gaming. Robustness mainly contains self-modification, distributional shift, robustness to adversaries, and safe exploration.

Many researchers have put forward some feasible solutions to AI safety problems. Some studies try to optimize reward functions (Amin et al., 2017; Krakovna et al., 2018). Some studies attempt to make agents learn from humans (Frye and Feige, 2019; Srinivasan et al., 2020). Others propose constrained RL for safe exploration (Achiam et al., 2017; Ray et al., 2019). We do not attempt to optimize the agent model, while the approach in this article is to avoid safety risks through the help of others. This is a new perspective for solving AI safety problems. The advantage of this model is that it can help artificial agents avoid risks and possibly help humans avoid some safety risks in the future.

### 2.2. Computational Models of ToM

The purpose of this article is to make agents understand others' false beliefs and policies in the environment through the ToM-SNN model and take assistance measures when other agents encounter danger in the environment. In this section, we summarize the previous methods of modeling ToM.

Baker proposed the Bayesian ToM (BToM) model, which modeled belief as the probability of an agent in a specific state (Baker et al., 2011). Based on this, dynamic Bayes net (DBN) can predict the target of an agent in the environment, which is the same as human's actual prediction (Baker et al., 2017). The reference of Baker's study lies in his symbolization of abstract terms in ToM, such as belief and desire, which makes the model more interpretable. Rabinowitz et al. (2018) proposed a model of predicting agent behavior and goal in a grid environment based on meta-learning, and this model can avoid false beliefs. Inspired by this study, Chen et al. (2021) built an authentic environment in which the robot can predict trajectories of the other robot. The starting point of these two studies is the same. They both hope that the agent can predict the behavior of others when its perspective is different from others and avoid false beliefs. Compared with Chen et al. (2021)'s study using end-to-end deep neural networks, Rabinowitz et al. (2018)'s study adopted meta-learning and helped advance the progress on interpretable AI. These two works have in common the fact that the observers do not execute the behaviors themselves.

Shum et al. (2019) combined the two methods of general models for action understanding and game theoretical models of recurrent reasoning and proposed a model to infer agent behavior based on the relationship between agents. This article argues that there are cooperative and competitive relationships among agents, and agents can predict behavior by predicting the relationship between each other. Nguyen et al. (2020) also used the idea of inferring agent relations to predict others' behavior by inferring the relationship among agents. Lim et al. (2020)'s model can help other agents by estimating the goals of other agents and putting the goals of others into its planning model. The limitation of these two models is that they assume that there is a specific relationship between agents. In many cases, agents can have different goals or tasks, and there is no specific relationship.

Zeng et al. (2020)'s study proposed a brain-inspired ToM model, which distinguishes the perspectives of self and others and beliefs of self and others. The model enables robots to pass the false beliefs task and has solid biological interpretability. Their article also compares the experimental results with brain imaging results and behavioral results to reveal the ToM mechanism in the brain. Inspired by Zeng et al. (2020)'s study, we proposed the ToM-SNN model, which focuses on how to make agents avoid safety risks rather than to reveal the biological mechanism of ToM.

Winfield (2018) also established the robot's ToM model to realize the prediction of other agents' behavior and consequences. Their study is influenced by the theory theory (TT) and simulation theory (ST) when modeling others' models. This modeling method is also very enlightening. However, the experimental design of their study does not highlight that their model can solve the problem of conflict of perspective and reasoning belief. One of the reasons why ToM is different from prediction is that it can correctly distinguish self from other's perspectives and beliefs.

## 3. Methods

### 3.1. The Functional Connectome of ToM

**Figure 1:**
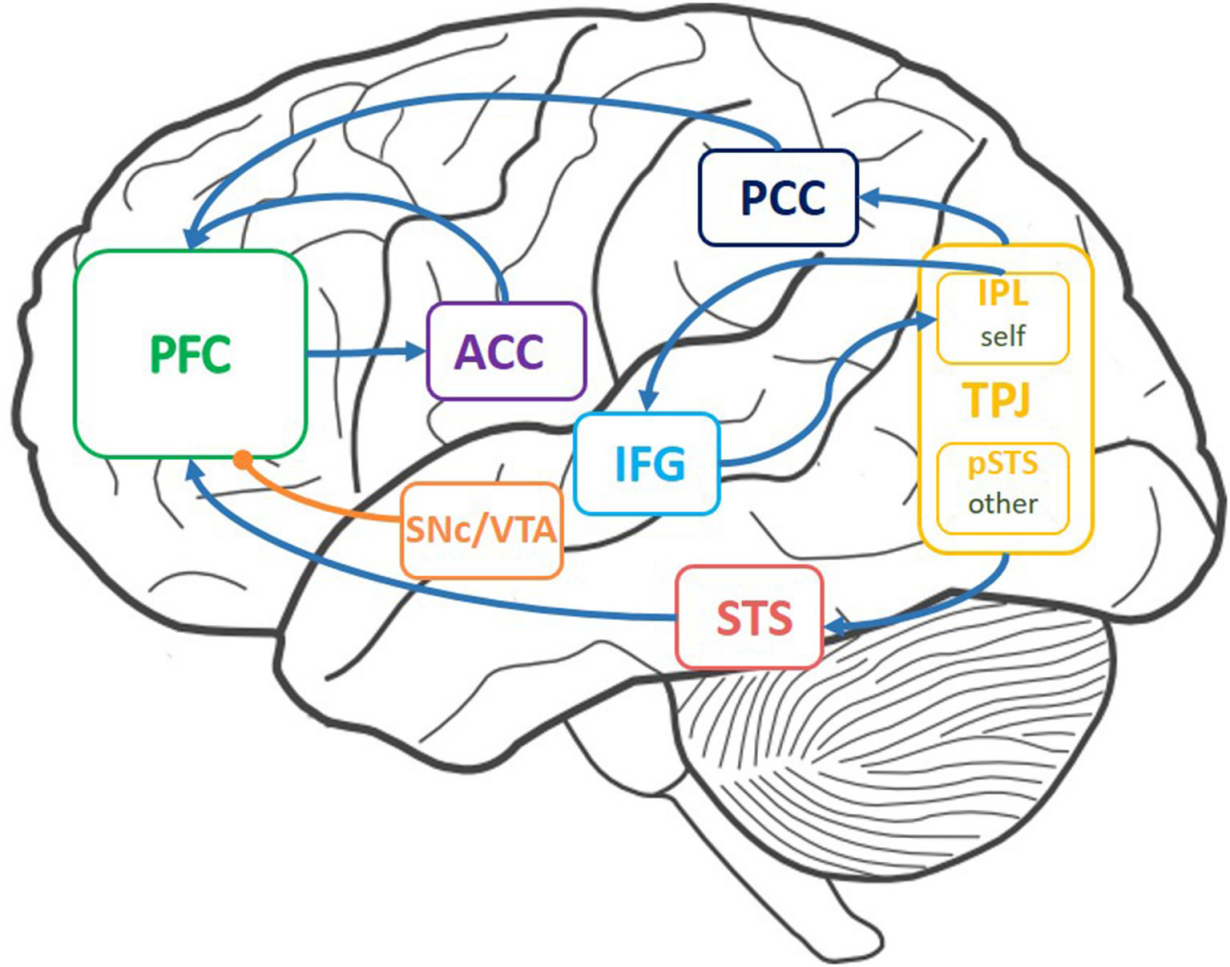
Brain areas involved in theory of mind (ToM) and the connections between these areas. These areas include the temporo-parietal junction (TPJ), the prefrontal cortex (PFC), the anterior cingulate cortex (ACC), the inferior frontal gyrus (IFG), and the substantia nigra pars compacta/ventral tegmental area (SNc/VTA). Arrows indicate connections between brain areas. The curve with a round head represents the projection path of dopamine.

According to our research, we drew a functional connectome of ToM with related brain areas (Abu-Akel and Shamay-Tsoory, 2011; Khalil et al., 2018; Zeng et al., 2020). The TPJ contains the IPL, which stores self-relevant stimuli, and the posterior superior temporal sulcus (pSTS), which stores other-relevant stimuli. The precuneus/posterior cingulate cortex (PCun/PCC) and the superior temporal sulcus (STS) send self-relevant, other-relevant information to the PFC. The PFC excites the ACC. The output of the ACC helps the PFC make decisions. The connection between the TPJ and the IFG is related to inferring others' false beliefs. Dopamine is projected to the PFC when humans make decisions or simulate others. Inspired by the neural circuits of ToM in the brain [concerning connections (Figure 1)] and functions [Table 1; (Abu-Akel and Shamay-Tsoory, 2011; Suzuki et al., 2012; Barbey et al., 2013; Koster-Hale and Saxe, 2013; Zeng et al., 2020)], we built the ToM-SNN model.

**Table 1:** The functions of brain areas.

**Brain area**	**Function**
TPJ	Perspective taking, stores mental states
IPL	Stores self-relevant mental states
pSTS	Stores other-relevant mental states
PCun/PCC	Sends self information
STS	Sends other information
ACC	Evaluates state value
PFC	Makes decisions, stimulates others' decisions
dlPFC	Stores working memory, predicts others' action
vmPFC	Infers others' behavior styles
IFG	Inhibits self-perspective
SNc/VTA	Is useful to elicit dopamine

### 3.2. The LIF Neuron Model

We use the Leaky Integrate-and-fire (LIF) model as the basic information processing unit of SNNs. The dynamic process of LIF neurons can be described by a differential function in Equation (1), where τ_*m*_ is the integral time delay constant of the membrane, and τ_*m*_ = *RC* where *R* is the membrane resistance and *C* is the membrane capacitor (Burkitt, 2006; Khalil et al., 2017). *I*_*i*_ denotes the input current of a neuron *i*. It can be seen that when the current continuously inputs to neuron *i*, the membrane potential begins to accumulate. When the membrane potential exceeds the threshold *V*_*th*_, the neuron will fire, and the membrane potential will be reset to *V*_*rest*_. In a period, the above phenomenon repeats continuously, and the neuron *i* continuously fire spikes. A spike train is a sequence of recorded times at which a neuron fires an action potential. Therefore, the neuron *i* will form a spike train *S*_*i*_.


(1) $$\begin{array}{l} \tau_{m}\frac{\text{d}V_{i}\left(t\right)}{\text{d}t}=-\left[V_{i}\left(t\right)-V_{rest}\right]+RI_{i}\left(t\right) \end{array}$$

### 3.3. Encoding and Decoding Schemes

Spiking neural networks need effective encoding methods to process the input stimulus and decoding methods to represent the output stimulus to handle various stimulus patterns. Population coding is “a method to represent stimuli by using the joint activities of a number of neurons. Experimental studies have revealed that this coding paradigm is widely used in the sensor and motor areas of the brain” (Wu et al., 2002). Besides, population coding tries to avoid the ambiguity of the messages carried within a single trial by each neuron (Panzeri et al., 2010).

**Encoding**. One requirement for encoding is to increase the difference among different input data. To alleviate it, we adopt population coding. In this article, the input data relates to the absolute and relative positions of agents. We use each neuron to represent a particular point on the horizontal or vertical axis.

**Decoding**. A requirement for decoding is to increase the representation precision of network output. Due to the randomness of the initial weights, the input current in the last layer is random. We use each neuron population to represent a particular output which enlarges the spatial domain and reduces the ambiguity of representation (Wu et al., 2019; Fang et al., 2021). By adopting a voting strategy and lateral inhibition, only one population of neurons fires among all populations, and it is regarded as the output.

### 3.4. Plasticity and Learning Model

We have chosen to implement biologically plausible STDP and R-STDP weight update rules to train the modules. Converging evidence about STDP indicates that synaptic weight changes are caused by the tight temporal correlations between presynaptic and postsynaptic spikes. STDP can be regarded as a temporary precision form of Hebbian synaptic plasticity because synaptic modification depends on the interspike interval within a critical window. When the presynaptic firing time is earlier than the postsynaptic firing time, the synapse between the two neurons will be enhanced, which is called long-term potential (LTP) (Δ*t* < 0), whereas reverse timing yields depression which is long-term depression (LTD) (Δ*t*> 0) (Kistler, 2002; Potjans et al., 2010; Héricé et al., 2016). A synaptic eligibility trace (*e*) stores a temporary memory of the relationship between the presynaptic neuron and postsynaptic neuron in a specific time window as shown in Equation (2) where *A*_±_ are learning rates, τ_±_ are STDP time constants, and Δ*t* = *t*_*pre*_ − *t*_*post*_ represents the delay between presynaptic spike arrival and postsynaptic firing.


(2) $$\begin{array}{l} \text{STDP}(\Delta t)=\{\begin{array}{cc} A_{+}\text{exp}[\Delta t/\tau_{+}] & \Delta t<0\\ -A_{-}\text{exp}[-\Delta t/\tau_{-}] & \Delta t>0 \end{array} \end{array}$$

In addition, reward-related dopamine signals can play the role of the neuromodulator that can help the brain learn by affecting synaptic plasticity. The eligibility trace can effectively bridge the temporal gap between the neural activity and the reward signals (Izhikevich, 2007; Frémaux and Gerstner, 2016; Mikaitis et al., 2018). The eligibility trace makes the synapses between neurons temporarily labeled, and then dopamine affects the labeled synapses. It is a transient memory which can be described in Equation (3) where τ_*e*_ is the time constant and δ is the Dirac delta function. Firings of presynaptic and postsynaptic neurons occur at times *t*_*pre*/*post*_, respectively. The weight change is based on the eligibility traces *e* and reward-related dopamine signals *r* shown in Equation (4). Inspired by dopamine, R-STDP is a learning method combining the advantages of STDP and is no longer unsupervised but more potent than STDP (Frémaux and Gerstner, 2016). R-STDP modulates network weights according to a synaptic eligibility trace *e* and a delayed reward *r*. Rewards represent reward-related dopamine signals and can be defined according to experiments. We described the reward function in section 4.1.


(3) $$\begin{array}{l} ė=-\frac{e}{\tau_{e}}+\text{STDP}\left(\Delta t\right)\delta \left(t-t_{pre/post}\right) \end{array}$$

(4) $$\begin{array}{l} ẇ=er \end{array}$$

### 3.5. The Architecture of the ToM-SNN

**Figure 2:**
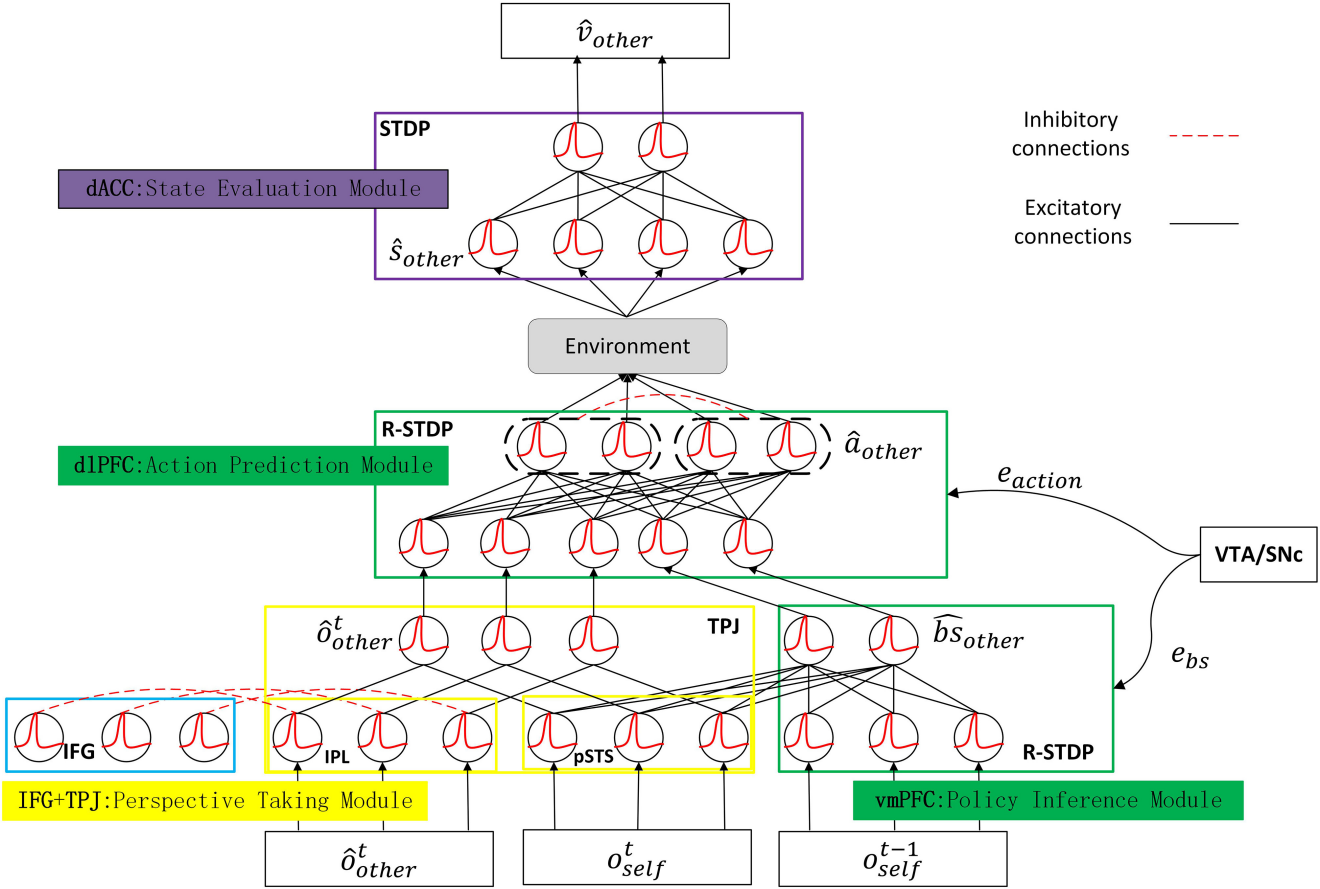
The architecture of the ToM-SNN model. The ToM-SNN model comprises the perspective taking module, the policy inference module, the action prediction module, and the state evaluation module, which are inspired by the TPJ, the vmPFC, the dlPFC, and the ACC, respectively. The perspective taking module parses current observation to form predictions about other's observation, ${ô}_{other}^{t}$. The policy inference module parses current observation and past observation to infer other's behavior style, ${\hat{bs}}_{other}$. The action prediction module parses predictions about other's observation and behavior styles to form other's predicted action, â_*other*_. When combining predictions about other's actions with environmental information, the agent can get other's next state, ŝ_*other*_. The state evaluation module parses other's next state to form state value, ${\hat{V}}_{other}$. The arrow in the figure indicates information transmission and does not involve the learning of network weight.

The ToM-SNN model incorporates the multiple brain area coordination mechanisms and is based on SNNs trained with STDP and R-STDP. We designed the ToM-SNN model shown in Figure 2 which shows the model structure, input, output, and training method. The ToM-SNN model is composed of the modules related to ToM: the perspective taking module, the policy inference module, the action prediction module, and the state evaluation module which are inspired by the TPJ, the vmPFC, the dlPFC, and the ACC, respectively. We trained the policy inference module and action prediction module by R-STDP. Dopamine is a neurotransmitter produced in the SNc and the VTA (Chinta and Andersen, 2005; Juarez Olguin et al., 2016). Research has shown that dopamine response is related to reward occurred and reward predicted (Schultz, 2007). Unexpected rewards increase dopaminergic neurons' activity, while the omission of expected rewards inhibits dopaminergic neurons' activity. Dopamine acts as a neuromodulator that affects synaptic plasticity. In the policy inference module and the action prediction module, error signals (*e*_*bs*_ and *e*_*action*_) just like dopamine modulates the synaptic weights based on Equation (4). Therefore, we trained these two modules with R-STDP.

Our model is a multiple brain areas coordination model composed of multiple modules. It is not an end-to-end multilayer neural network. The advantages of a multiple brain areas coordinationmodel are reflected in two aspects. First, inspired by brain structure and function, modules in the ToM-SNN corresponding to specific brain areas have specific functions. The end-to-end neural networks are “regularly described as opaque, uninterpretable black-boxes” (Rabinowitz et al., 2018). Our model is more biologically plausible and more interpretable. Second, a multiple brain areas coordination model can reduce the burden of training. When a new feature appears in the task, only the module for this feature needs to be retrained. So this structure can reduce the amount of calculation and improve efficiency. The policy inference module, the action prediction module, and the state evaluation module are fully connected SNNs with two layers. Details of the two-layers SNNs are as follows. The input current of the input layer and the output layer is denoted by *I^in^* and *I^out^*, respectively. The output spikes of the input layer and the output layer are denoted by *S^in^* and *S^out^*, respectively. Section 3.1 describes the neural spiking process. At each time step *t*, the input current to neuron *j* at the output layer is integrated as Equation (5).


(5) $$\begin{array}{l} I_{j}^{out}\left(t\right)=\underset{i}{\sum }w_{ji}S_{i}^{in}\left(t\right) \end{array}$$

The *w*_*ji*_ denotes the synaptic weight. The $I_{j}^{out}\left(t\right)$ can change neuron *j*'s membrane potential $V_{j}^{out}\left(t\right)$ as shown in Equation (6) and the neuron *j* generates an output spike at time *t* ($S_{j}^{out}\left(t\right)$) when $V_{j}^{out}\left(t\right)$ crosses the threshold *V*_*th*_ as shown in Equation (7).


(6) $$\begin{array}{l} V_{j}^{out}\left(t\right)=V_{j}^{out}\left(t-1\right)+\frac{dt}{\tau_{m}}\left[-V_{j}^{out}\left(t-1\right)+V_{rest}+RI_{j}^{out}\left(t\right)\right] \end{array}$$

(7) $$\begin{array}{l} S_{j}^{out}(t)=\Theta (V_{j}^{out}(t)-V_{th})\text{ with }\Theta (x)=\{\begin{array}{cc} 1, & \text{if }x\geq 0\\ 0, & \text{else} \end{array} \end{array}$$

The total number of spikes *c*^*p*^ generated by the neuron population *p* can be determined by summing all neurons' spikes in the population *p* over the simulation period *T* as Equation (8). Each population has *J* neurons. The population *p*_*max*_ that sent the most spikes is selected as the output as shown in Equation (9). *P* is the number of populations.


(8) $$\begin{array}{l} c^{p}=\overset{J}{\underset{j}{\sum }}\overset{T}{\underset{t}{\sum }}S_{j}^{out}\left(t\right) \end{array}$$

(9) $$\begin{array}{l} p_{max}=argmax\left(c^{0},c^{1},\ldots ,c^{P}\right) \end{array}$$

The PFC receives numerous dopaminergic projections. Dopamine affects synaptic plasticity. The reward can regulate the weight through R-STDP. A positive reward is exploited at the synaptic level to reinforce the correct sequence of actions, whereas a negative reward weakens the wrong. When we model modules related to the PFC, we train the model with R-STDP. We use the STDP mechanism to modulate the network learning process in the ACC.

We will describe these modules and the parameters involved in them in detail in the following paragraphs.

**Perspective taking module**. An essential ability of ToM is to distinguish between different perspectives in the same situation simultaneously. In the reasoning about others' beliefs, the conflict between self-and-other perspectives in the TPJ will activate the IFG, then IFG will inhibit self-relevant stimuli in the IPL. Inspired by this, we used the IFG module presented by the Brain-ToM (Zeng et al., 2020) to inhibit self-relevant stimuli. The input of the module consists of two parts: self-relevant stimuli and other-relevant stimuli. The output is an inference about other people's observations (ô_*other*_). The weights of the connections between the IFG and the TPJ remain unchanged in this model.

**Policy inference module**. This module is used to model the function of the vmPFC brain area to distinguish the behavior styles. We preprocessed the self-observation *o*_*self*_ composed of visible agents' positions at time *t* and at time *t* − 1 and then input them into the model. The output is the agent's behavior styles denoted by ${\hat{bs}}_{other}$. The output is the policy characteristics corresponding to the population which fired the most spikes. The vmPFC receives numerous dopaminergic projections. The research has shown that dopamine response is related to reward occurred and reward predicted (Schultz, 2007). The weights between the two layers are equivalent to synapses. We denote the predicted other's behavior styles error by *e*_*bs*_ shown in Equation (10). The error will regulate synaptic plasticity in the form of dopamine based on Equation (4). Therefore, *e*_*bs*_ is regarded as a reward when we trained our module with R-STDP. γ and β are constants.


(10) $$\begin{array}{l} e_{bs}=-|{\hat{bs}}_{other}-bs_{other}|*\gamma +\beta \end{array}$$

**Action prediction module**. This module is used to model the function of the dlPFC brain area to predict others' behaviors. The input is formed by concatenating the predicted other's observation, ô_*other*_ generated by the perspective taking module with its behavior style, ${\hat{bs}}_{other}$. The output is the predicted others' action, â_*other*_. We denoted the predicted other's action error by *e*_*action*_ shown in Equation (11) where *a*_*other*_ is the actual action. The error regarding as a reward can be used to regulate the weight through R-STDP. A positive reward is exploited at the synaptic level to reinforce the correct sequence of actions, whereas a negative reward weakens the wrong. We will describe the training process in detail in section 4.2. The predicted other's action error is regarded as a reward to modulate weights. Besides, different populations will inhibit each other, and neurons in the same population do not inhibit each other. As shown in Figure 2, the black dashed box represents a population. In the output layer in this module, lateral inhibition between populations of neurons reduces the activity of exited populations' neighbors.


(11) $$\begin{array}{l} e_{action}=\{\begin{array}{cc} 1, & \text{if }{\hat{a}}_{other}==a_{other}\\ -1, & \text{else} \end{array} \end{array}$$

**State evaluation module**. This module is used to model the function of the ACC brain area. The goal of the state evaluation module is to evaluate the safety of the observed agent. The input is the predicted state of others denoted by ŝ_*other*_ which is formed by the predicted others' action, â_*other*_ output is other agents' safety status denoted by $\hat{v}$. Because there are two kinds of safety status: safe and unsafe, the STDP mechanism can perform well.

**Figure 3:**
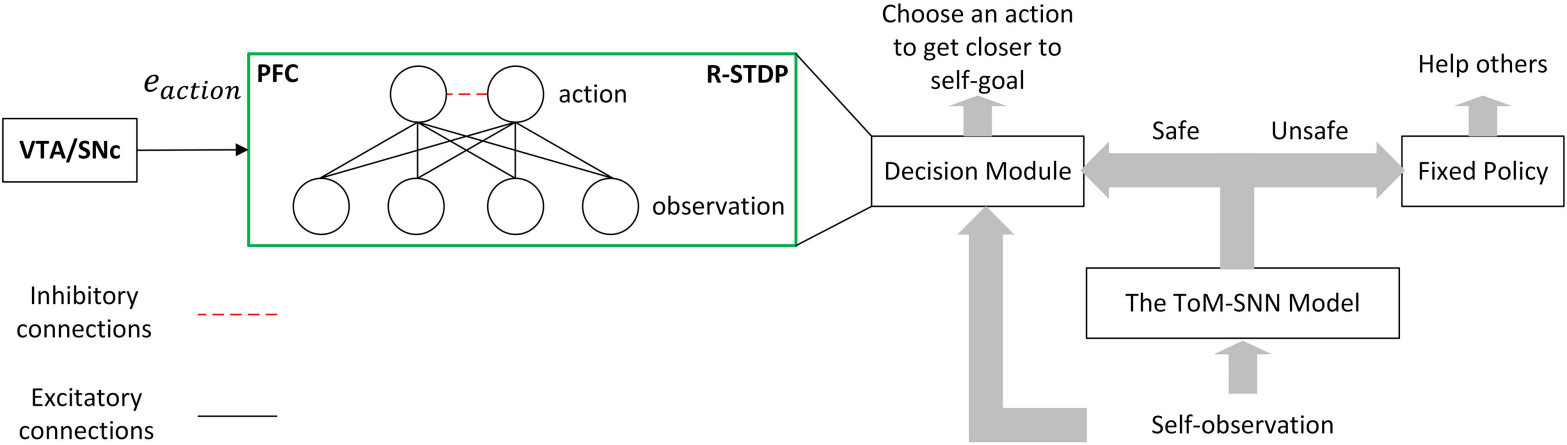
Simple architecture for reducing the safety risks of others. The ToM-SNN model can infer others' safety status by inferring their beliefs and behavior styles. The agent will continue carrying out its task when others are in a safe situation, whereas it will help with fixed policy when others are unsafe. The arrow in the figure indicates information transmission and does not involve the learning of network weight.

After introducing the ToM-SNN model, we design a simple architecture so that the agent can take practical measures to reduce others' safety risks when inferring other agents' unsafe status. The agent can choose an action to get closer to its own goal when it infers others safe. This process is shown in Figure 3.

**Decision module**. The decision model in Figure 3 shows the input, output, and training methods. This module is designed to learn a policy to make agents arrive at their goals. The input of the module is the observation *o*_*self*_. The output of the module is the action *a*_*self*_. In the output layer, lateral inhibition will also reduce the activity of exited populations' neighbors. The decision module is trained by R-STDP. The left part of the figure shows how the VTA/SNc sends dopamine to the PFC. Dopamine acts on the synapses of the two layers of neurons in the decision module to help update the network weight. More specifically, the reward plays the role of dopamine, and it is combined with the eligibility traces, which can modulate the weights based on Equation (4). In the process of interacting with the environment, an agent adjusts its policy according to the reward. The reward function is defined in section 4.1.

## 4. Experiments

Our main goal is that an agent can infer others' safety status with the ToM-SNN model and choose to interfere when necessary. An agent can unconsciously expose itself to potentially unsafe situations due to holding either false beliefs of its states or bad policies. This section tries to verify that the ToM-SNN model can find others' potentially unsafe situations by introducing experimental environments, model training, experimental method, experiments, and results.

### 4.1. Environments and Agents' Policies

**Figure 4:**
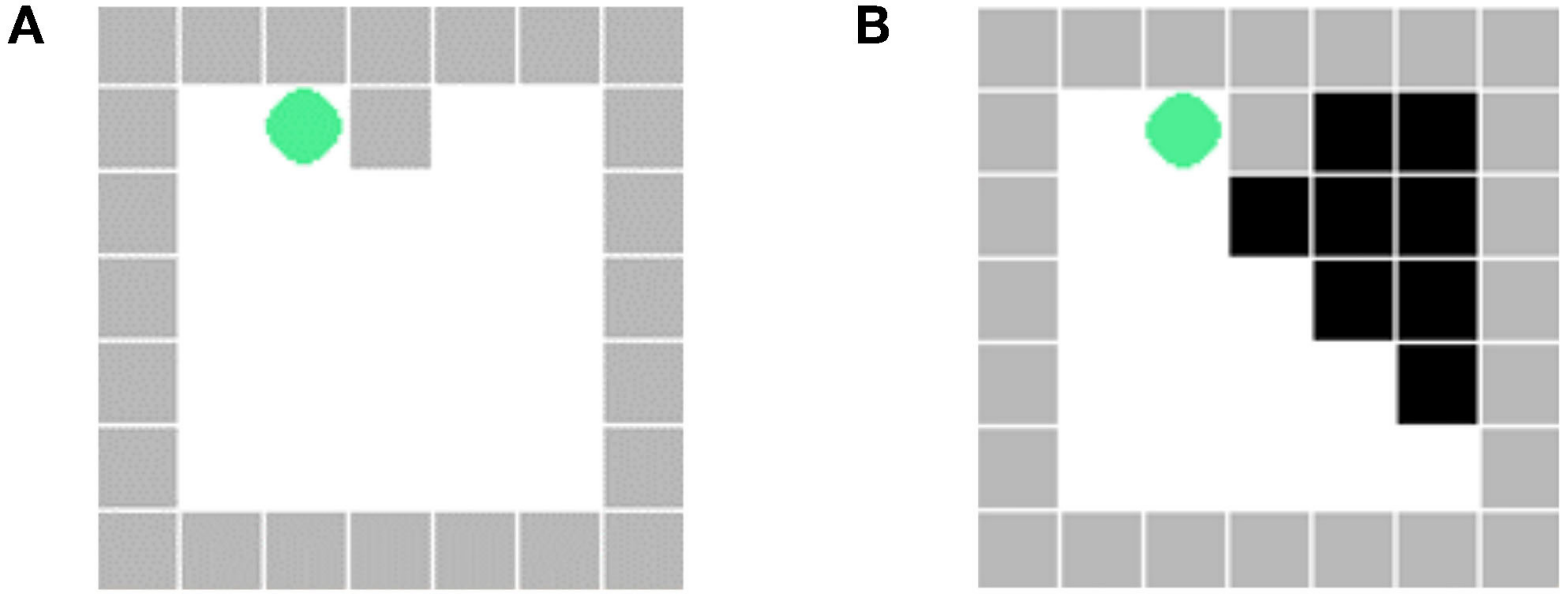
**(A)** The actual position of the agent. **(B)** The view of the agent at the current position. The black part is the area blocked by the wall, which is invisible to the agent.

To verify the effectiveness of the ToM-SNN model, we conducted various experiments in the gridworld environments with random agents' starting positions and random blocking walls. The gridworld environment is implemented with PyGame. The experimental environment is a 7 × 7 gridworld with a common action space(up/down/left/right/stay), goals, and random blocking walls. The wall will block part of the view of an agent in the environment shown in Figure 4. The visible area of an agent is the white area in Figure 4B. The environments are fully observable for agents if no wall blocks their views.

We designed three different kinds of policies for agents: the reckless policy, the experienced policy, and the cautious policy.

When an agent is taking the reckless policy, it does not consider the impact of their behaviors on other agents. The reward is only related to their distance from the goal. The reward function is shown in Equation (12). *Dp*_*t*_, *Dp*_*t* − 1_ is the Euclidean distance between the current position and the goal at time *t*, *t* − 1, respectively. According to Equation (12), when the agent gets further away from the goal, it will get a negative reward.


(12) $$\begin{array}{l} r=\frac{Dp_{t-1}-Dp_{t}}{Dp_{t-1}} \end{array}$$

An agent with experienced policy learns a safe strategy without colliding with other agents and walls. The reward is related to the goal and collision. The experienced agents will get a negative reward when colliding with others. This kind of agent can actively avoid others that can be observed. The reward function is shown in Equation (13).


(13) $$\begin{array}{l} r=\{\begin{array}{c} \frac{Dp_{t-1}-Dp_{t}}{Dp_{t-1}}\\ -5,\text{ if collision} \end{array} \end{array}$$

The third kind of policy is cautious. Since the wall will block the perspective and make the agent have a false belief in the state, the agent will tend to take action away from the walls. The reward function is shown in Equation (14). *Dw*_*t*_, *Dw*_*t* − 1_ is the Euclidean distance between the current position and the wall at time *t*, *t* − 1, respectively.


(14) $$\begin{array}{l} r=\{\begin{array}{c} \frac{Dp_{t-1}-Dp_{t}}{Dp_{t-1}}\\ \frac{Dw_{t}-Dw_{t-1}}{Dw_{t-1}},\text{ if}\\ -5,\text{ if collision} \end{array}\;Dw_{t-1}<=1-5, \end{array}$$

The three kinds of agents adopt the decision module in the left part of Figure 3. The input of the model is the observation, including the location of the goal, the location of walls, and the location of other agents. We used population coding to encode the observation, which is represented by [7·7·(4+8)] neurons, where (7·7) is the size of the gridworld, 4 is the number of walls' features and 8 is the number of other agents' features. The decision module is trained by R-STDP, and the reward can be obtained by the reward function shown in Equations (12)–(14). After training the decision modules, we get three kinds of agents with fixed policies.

### 4.2. Model Training

In this subsection, we describe the model parameters and the training of the networks. Resting potentials are around -70 mV (Brette, 2006; Chen and Jasnow, 2011). For the hyper-parameters of the LIF neuron as described in section 3.2, we set *V*_*th*_ = −55 mV, *V*_*rest*_ = −75 mV, τ_*m*_ = 20 ms according to the research. In the experiment, we simplify the process of depolarization and repolarization. For the hyper-parameters of the STDP and R-STDP as described in Section 3.4, we set *A*_+_ = 0.925, *A*_−_ = 0.1 (Kistler, 2002), τ_*e*_ = 5 ms, τ_+_ = τ_−_ = 20 ms (Friedmann et al., 2013) according to the research. The synaptic efficacy is increased if presynaptic spikes arrive slightly before the postsynaptic firing and the synapse is weakened if presynaptic spikes arrive a few milliseconds after the output spike. For the hyper-parameters of the ToM-SNN model as described in section 3.5, we set γ = 2, β = 1 to make sure the parameter, *e*_*bs*_, belongs to the closed interval [−1, 1] and speed up the convergence of the network by normalizing weights to the closed interval [−1, 1].

We trained our model to predict the safety status of others based on the policies of different agents in random environments with either two agents or one agent and walls with 300 episodes. An episode process is that the agents start at the starting position until all agents end the game.

The number of neurons in different modules is listed in Table 2. The observed states are encoded by populations with [7·7·(4+8)] neurons where (7·7) is the size of the gridworld, 4 is the number of walls' feature and 8 is the number of other agents' features. The input of the policy inference module is encoded by (6+8) neurons where 6 is the number of agents' policy characteristics which the policy inference module predicted at time *t* − 1 and another 8 neurons encode the difference of perspective and others' safety status. The characteristics of agents' policies are encoded by one population with 6 neurons and we used (3·6) neurons to represent three kinds of policies. The input of the action prediction module is composed of other agents' observed states and their policy characteristics. The action is encoded by one population with 6 neurons and 5 populations can represent all actions. When the output of the action prediction model is different from the behavior of other agents, the model will receive a negative reward. On the contrary, if the prediction is correct, it will get a positive reward. The reward will help adjust the weight of the network. The input of the state evaluation module is other's state at time *t* + 1, which is got by combining collected data about environment information with a prediction about other's action at time *t*. The output of the state evaluation module is composed of two safety status, which is encoded by two population with 6 neurons. When there is a collision, neurons associated with characterizing risk will fire. Combined with the flow in the right half of block Figure 3, if others are safe in the next state, the bystander will not help. Otherwise, the bystander will prevent the behavior of other agents.

**Table 2:** The number of neurons in different modules.

**Modules**	**Number of neurons in input layer**	**Number of neurons in output layer**
Perspective taking module	7·7·(4+8)·2	7 ·7·(4+8)
Policy inference module	(6+8)	3·6
Action prediction module	7·7·(4+8)·3	5·6
State evaluation module	7·7·(4+8)	2·6

### 4.3. Experiments and Results

In the first two subsections, first, we introduced the random environments and three kinds of policies. Then, we introduced the training process of the ToM-SNN model. In this section, we applied the ToM-SNN model to the bystander and tested it in the random gridworld environments. Additionally, we compared the performance of the agents and the safety situation when the bystander did not use the ToM-SNN model. Based on the following experiments, we show that the bystander with the ToM-SNN model can help others avoid risks in many random environments when necessary.

**Figure 5:**
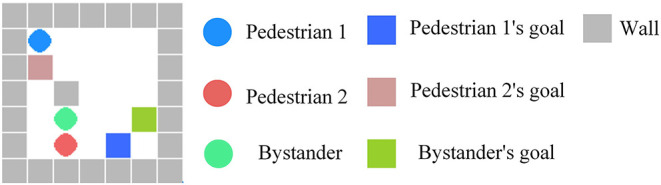
An example of a random environment. The environment consists of three agents: a bystander and two pedestrians. Different agents have different goals to reach. Agents cannot pass through walls.

A false belief task is a type of task used in ToM studies in which subjects must infer that another person does not possess knowledge that they possess. Inspired by this experimental paradigm, we designed the experiment. The feature of the experimental scene is to make agents possess some different knowledge. The occlusion of the wall will make the agents in different locations observe the environment differently. Agents with different initial strategies will choose different behaviors in the process of executing tasks. There are three agents in potentially risky environments. We randomized agents' starting positions and blocking walls in the environments (e.g., Figure 5). Random combinations of agents with different starting positions and random walls will increase the randomness of the environment. Besides, the shape of walls and the starting positions in the test environments are not exactly the same as those in the training environments. In each episode, we fixed the policy of pedestrian 1 and the bystander to reckless. We conducted 100 random experiments, respectively, when the policy of pedestrian 2 is reckless, cautious, and experienced and the bystander does not know the policy characteristics of pedestrian 2. When all agents reach their goals, one episode will end. When the bystander predicts the risks of others, it will stop others from taking action to move on. At the same time, helping others makes the bystander lose scores.

We give the agent an initial performance score, setting *R*_*base*_ to 50 and setting *L*_*collision*_ to 40 if the agent collides with others and to 0 otherwise. The performance score will be consumed as time passes. If the agent consumes time *t* to reach the goal, the performance score is the number of points subtracted from *R*_*base*_ by *C*_*time*_ · *t*. We set *C*_*time*_ to 3. We set that helping others will reduce performance scores. We set *L*_*help*_ to 10 if the agent helps others and to 0 otherwise. The final performance score is no less than zero shown in Equation (15). The risk assessment can be understood as that collision causes risks.


(15) $$\begin{array}{c} P=max\left(R_{base}-C_{time}\cdot t-L_{collision}-L_{help},0\right) \end{array}$$

In the following, we conducted comparative experiments with and without the ToM-SNN model. We analyzed the results of the compared experiments by using performance scores and risk assessment.

**Figure 6:**
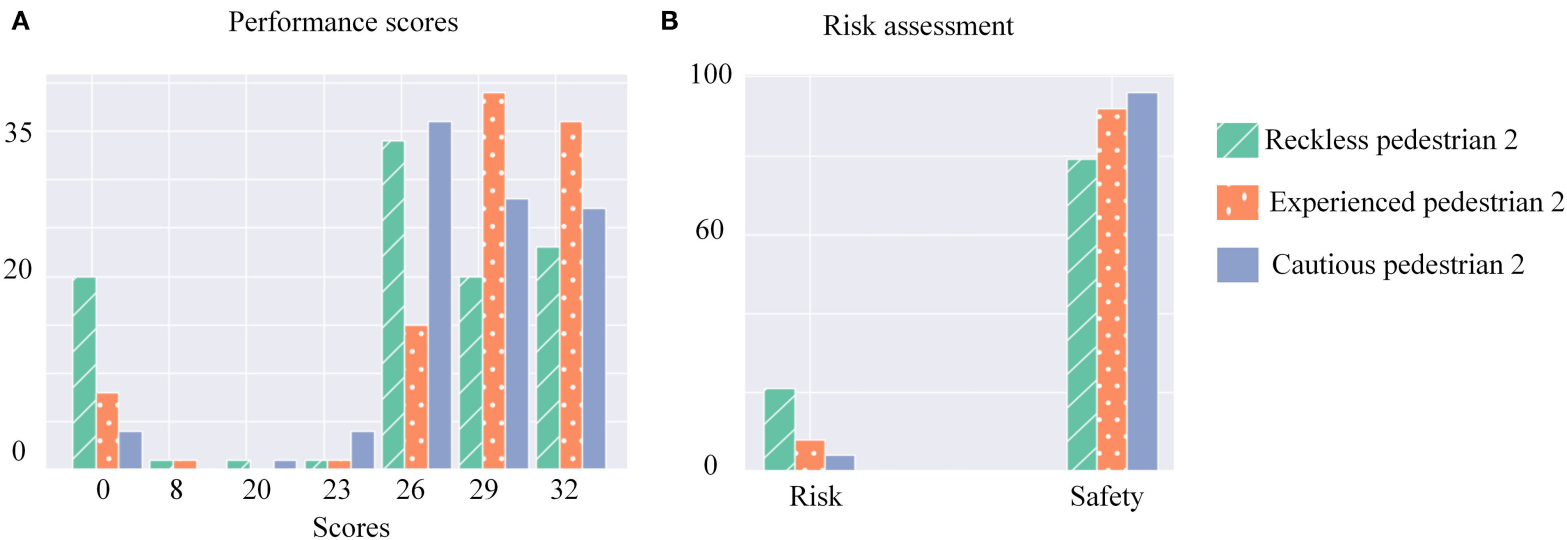
**(A)** Histograms of three kinds of pedestrian 2's performance scores. The horizontal axis represents performance scores, and the vertical axis represents the number of occurrences of scores. **(B)** Histograms of three kinds of pedestrian 2's risks assessment. The horizontal axis represents the two states of risk and safety, and the vertical axis represents the number of occurrences of the different states.

First, we conducted experiments without the ToM-SNN model and assessed the performance and risks of pedestrian 2 with different policies shown in Figure 6. It can be seen that cautious agents have a small probability of encountering risks but have fewer opportunities to get higher scores than experienced agents. Reckless agents will take risks and increase benefits. The probability of reckless agents getting high scores is significantly lower than the others, but they can also reach their goals safely sometimes because the environments are random, and not all environments have risks.

Second, we endowed the bystander with the ToM-SNN model. To explore the effect of the ToM-SNN model on agents with different policies in a risky environment, we assessed the performance and risks of pedestrian 2 with different policies.

**Figure 7:**
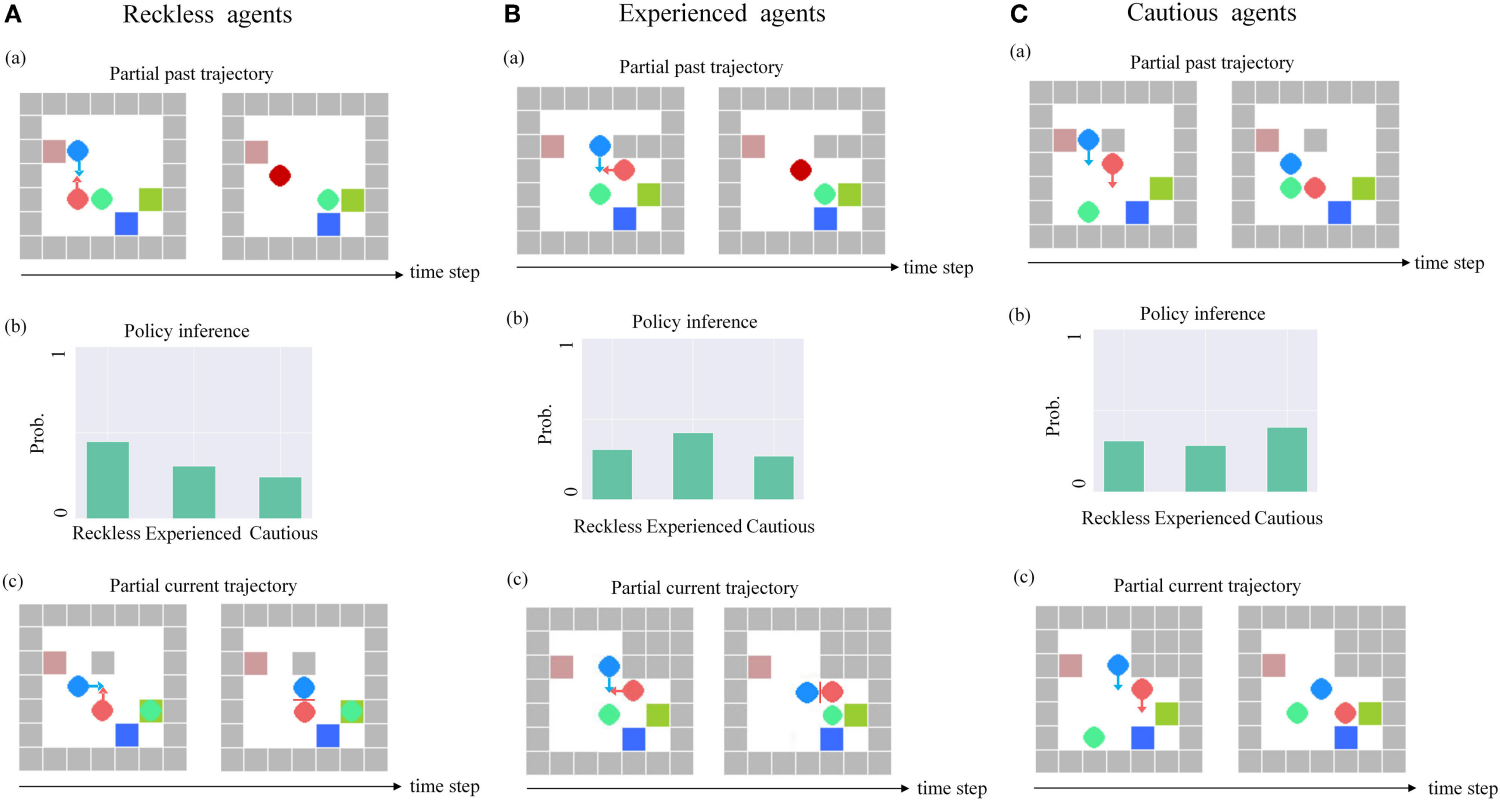
The ToM-SNN model on three kinds of agents in random environments. **(A)** The bystander with the ToM-SNN model's prediction of pedestrian 2's policy characteristic based on the partial past trajectory [e.g., **A**(a)] is shown in **A**(b). In another environment, the bystander with the ToM-SNN model can predict the risks of pedestrian 2 and help it to avoid risks. **(B)** The bystander with the ToM-SNN model predicts that pedestrian 2's policy characteristic is experienced based on the partial past trajectory [e.g., **B**(a)] is shown in Figure. In another environment, the bystander with the ToM-SNN model can predict the risks of pedestrian 2 and help it to avoid risks. **(C)** The bystander with the ToM-SNN model predicts that pedestrian 2 is a cautious agent shown in **C**(b) based on the partial past trajectory [e.g., **C**(a)]. In another environment, the bystander with the ToM-SNN model did not interfere with pedestrian 2 's action because it evaluated pedestrian 2's next state was safe [**C**(c)].

**Pedestrian 2 with the reckless policy**. We explored the impact of the ToM-SNN model when pedestrian 2 is reckless. The ToM-SNN model predicts pedestrian 2's policy characteristics based on partial past trajectory [e.g., Figure 7A(a)]. According to the experimental phenomenon (e.g., Figure 7A), we found that the bystander with the ToM-SNN model can predict pedestrian 2's actions. If it predicts that pedestrian 2 will be in danger, the bystander will take help strategies to help pedestrian 2 avoid risks.

**Pedestrian 2 with the experienced policy**. We focused on pedestrian 2 with experienced policies. In some random environments, the random walls just block the perspective of pedestrian 2 [e.g., Figure 7B(a)], which causes it wrong decisions. Figure 7B(b) shows the bystander with the ToM-SNN model can infer the policy characteristic of pedestrian 2 based on the partial past trajectory, infer pedestrian 2 false belief in environments, and predict experienced agents can take the wrong action. Figure 7B(c) shows the bystander help it to avoid risks.

**Pedestrian 2 with the cautious policy**. In the same test environment, the bystander inferred that pedestrian 2 is a cautious agent shown in Figure 7(b). Although the walls blocked cautious agents' perspective, the bystander can predict that the cautious agents will walk away from the wall based on partial past trajectory [e.g., Figure 7C(a)]. Therefore, the bystander's next state assessment of the cautious agent is safe, so the bystander will not help [e.g., Figure 7C(c)].

**Figure 8:**
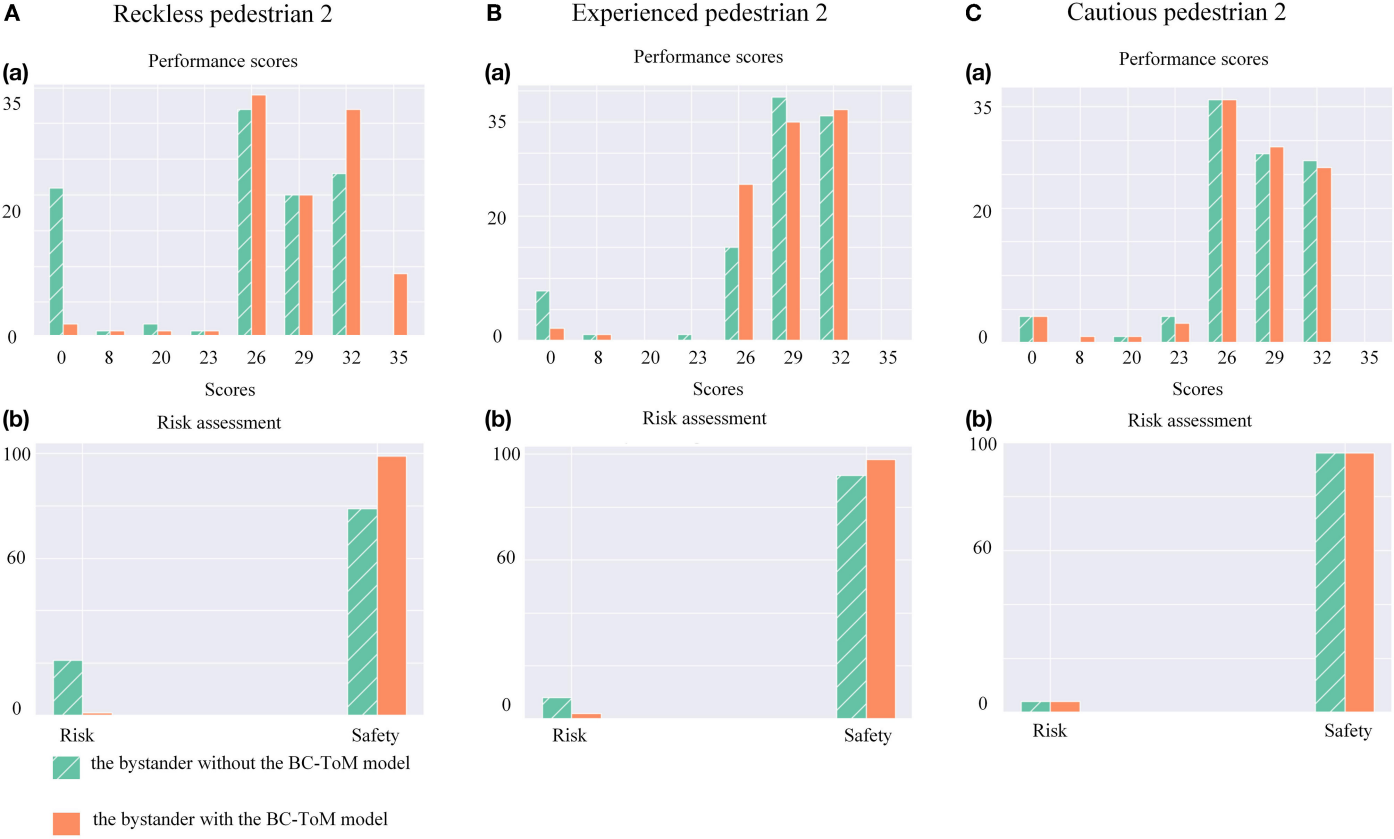
**(A)**(a) Histograms of reckless agents' performance scores. (b) Histograms of reckless agents' risks assessment. **(B)**(a) Histograms of experienced agents' performance scores. (b) Histograms of experienced agents' risks assessment. **(C)**(a) Histograms of cautious agents' performance scores. (b) Histograms of cautious agents' risks assessment.

Based on the experimental phenomenon, we proved that the ToM-SNN model could infer other's false beliefs, policy characteristics, predict other's actions and evaluate other's safety status. Then we analyzed the effect of having the ToM-SNN model and not having the ToM-SNN model on agents with different policies shown in Figure 8.

First, we analyzed the first row of Figure 8, the performance scores. The figure shows that the scores of pedestrian 2 with three kinds of policies are mainly distributed in the region of 0 point and more than 23 points. The distribution of high score regions (*score* ≥ 23) indicates that the agents can almost reach the goal in less than eight time steps ((50−26)/3) without risk. According to Equation (15), it can be found that when the agents collide with others, they will get zero points. The rest of the scores within the interval of 8 to 23 indicate the agents' poor performance in random environments. The figure can show that the ToM-SNN model reduces the number of scores of 0 for reckless and experienced agents and increases the number of cases of high scores for reckless agents. The reckless pedestrian 2 takes risks and increases benefits. Then, we analyzed the second row of Figure 8, the risk assessment. From the figure, it can be seen that the ToM-SNN model clearly reduces the risk of reckless and experienced agents but almost has no effect on pedestrian 2 with cautious policy. Because the ToM-SNN model can determine that pedestrian 2 is cautious and does not encounter risk in the environment based on the observation of pedestrian 2. The bystander with the ToM-SNN model will not help it. Since cautious policies can perform erratically in random environments sometimes, the risk of cautious agents can be ignored when evaluating the ToM-SNN model performance.

As mentioned above, helping others will affect the bystander performance scores. We counted the scores of the bystander, respectively, when pedestrian 2 is reckless, experienced, and cautious, and showed the average value, variance, and minimum value of the scores in Table 3. This shows that the ToM-SNN model can not only help others avoid risks, but also choose different behaviors for different agents, so as to reduce their own losses.

**Table 3:** The bystander's performance scores.

**The policy of pedestrian 2**	**Reckless**	**Experienced**	**Cautious**
Performance scores	35.93 ± 3.83	37.28 ± 2.42	37.76 ± 0.82

## 5. Discussion and Conclusion

We proposed a new idea of using the ToM-SNN model to help other agents avoid safety risks. The ToM-SNN model is combined with bio-inspired SNNs modeled multi-brain areas which mainly include the TPJ, part of the PFC, the ACC, and the IFG. The experimental results show that the ToM-SNN model can infer others' policy characteristics, predict the behavior of others, assess others' safety status and, thus, reduce others' risks. In addition, the model is rational. Even in the same potentially risky environment, the model will behave differently for agents with different policies so as to help others as much as possible while minimizing their own losses. More importantly, the structure and learning mechanism of the model are inspired by the ToM loops in the biological brain, and the input and output of the network have meanings, which makes the model more biologically interpretable. That is to say, our model is an interpretable, biologically plausible model which can avoid safety risks.

We focus on building a brain-inspired theory of mind spiking neural network model to distinguish different agents, predict others' actions and evaluate their safety. We successfully build a ToM spiking neural network model to avoid safety risks for the first time. Although Zeng et al. (2020) have established the Brain-ToM model, this model is inclined to reveal the biological mechanism of ToM in the brain. On the basis of it, our model added the policy inference module and action prediction module and combined with the decision-making system. We tried to use the ToM-SNN model to avoid safety risks. Compared with the Brain-ToM model from Zeng et al. (2020): (1) The ToM-SNN model can simulate others' decisions in combination with others' behavior styles, whereas the agent with the Brain-ToM can only infer other agents that are the same as itself. Besides, our model can infer others' current beliefs and predict others' decisions and safety status. Different agents will have different behaviors in the same task due to different policies. Therefore, predicting others' actions according to their policies is important. We use the experiment in section 4 to test the Brain-ToM model. It can be seen from Table 4 that the Brain-ToM model performs almost the same for three types of agents. The results show that the Brain-ToM model can not produce different feedback for agents with different policies. (2) In the process of simulating others' decisions, dopamine helps the PFC predict others' decisions. Therefore, our model uses the R-STDP to train the policy inference module and the action prediction module to get the appropriate weights, whereas the weights of the PFC part in the Brain-ToM remain unchanged. (3) We try to solve others' safety problems through the ToM. The agent with the ToM-SNN model helps others avoid safety risks successfully. Additionally, their work provides a possible computational model and hints on how infant infers and understands other people's beliefs. The two models are based on SNNs through brain-inspired mechanisms and have contributions to the ToM models.

**Table 4:** Compared bystander's performance scores.

**The policy of pedestrian 2**	**Reckless**	**Experienced**	**Cautious**
Performance scores (the ToM-SNN model)	35.93 ± 3.83	37.28 ± 2.42	37.76 ± 0.82
Performance scores (the Brain-ToM model)	36.00 ± 3.74	36.18 ± 3.77	36.26 ± 3.58

There is much work to do to scale the ToM-SNN model. First, we focus on building the ToM model to help others avoid safety risks. In the future, we hope to be inspired by the mirror neuron system and establish a biologically plausible model to understand others' actions (Khalil et al., 2018). In addition, a vital point of this article is that the ToM model can influence the decision-making process so as to help reduce safety risks. The safety risk studied in this article is still relatively single, and there is only one risk at a time in the experiment. In the future, we hope to improve the model to a social decision-making model such as making moral uncertainty reach intuitively reasonable trade-offs between ethical theories (Ecoffet and Lehman, 2021).

## Data Availability Statement

The raw data supporting the conclusions of this article will be made available by the authors, without undue reservation.

## Author Contributions

ZZ, EL, and YZe designed the study. ZZ and FZ performed the experiments and the analyses. EL and YZe were involved in problem definition and result analysis. ZZ, EL, FZ, YZe, and YZh wrote the manuscript. All authors contributed to the article and approved the submitted version.

## Funding

This study was supported by the National Key Research and Development Program (Grant No. 2020AAA0104305), the Strategic Priority Research Program of the Chinese Academy of Sciences (Grant No. XDB32070100), the National Natural Science Foundation of China (Grant No. 62106261), and the Beijing Academy of Artificial Intelligence (BAAI).

## Conflict of Interest

The authors declare that the research was conducted in the absence of any commercial or financial relationships that could be construed as a potential conflict of interest.

## Publisher's Note

All claims expressed in this article are solely those of the authors and do not necessarily represent those of their affiliated organizations, or those of the publisher, the editors and the reviewers. Any product that may be evaluated in this article, or claim that may be made by its manufacturer, is not guaranteed or endorsed by the publisher.
