# Corrigendum: A Brain-Inspired Theory of Mind Spiking Neural Network for Reducing Safety Risks of Other Agents

**DOI:** 10.3389/fnins.2022.920292

**Published:** 2022-05-19

**Authors:** Zhuoya Zhao, Enmeng Lu, Feifei Zhao, Yi Zeng, Yuxuan Zhao

**Affiliations:** ^1^Research Center for Brain-inspired Intelligence, Institute of Automation, Chinese Academy of Sciences, Beijing, China; ^2^School of Future Technology, University of Chinese Academy of Sciences, Beijing, China; ^3^School of Artificial Intelligence, University of Chinese Academy of Sciences, Beijing, China; ^4^National Laboratory of Pattern Recognition, Institute of Automation, Chinese Academy of Sciences, Beijing, China; ^5^Center for Excellence in Brain Science and Intelligence Technology, Chinese Academy of Sciences, Shanghai, China

**Keywords:** brain-inspired model, safety risks, SNNs, R-STDP, theory of mind

In the original article **Wu et al. (2002)** was not cited in the article. The citation has now been inserted in ***Methods, Encoding and Decoding Schemes, Paragraph 1*** and should read:

**Spiking neural networks need effective encoding methods to process the input stimulus and decoding methods to represent the output stimulus to handle various stimulus patterns. Population coding is “a method to represent stimuli by using the joint activities of a number of neurons. Experimental studies have revealed that this coding paradigm is widely used in the sensor and motor areas of the brain” (Wu et al., 2002). Besides, population coding tries to avoid the ambiguity of the messages carried within a single trial by each neuron (Panzeri et al., 2010)**.

Additionally, in the original article, **Rabinowitz et al. (2018)** should be referenced more than once. The citation has now been inserted in ***Methods, The Architecture of the ToM-SNN, Paragraph 2*** and should read:

**Our model is a multiple brain areas coordination model composed of multiple modules. It is not an end-to-end multilayer neural network. The advantages of a multiple brain areas coordinationmodel are reflected in two aspects. First, inspired by brain structure and function, modules in the ToM-SNN corresponding to specific brain areas have specific functions. The end-to-end neural networks are “regularly described as opaque, uninterpretable black-boxes” (Rabinowitz et al., 2018). Our model is more biologically plausible and more interpretable. Second, a multiple brain areas coordination model can reduce the burden of training. When a new feature appears in the task, only the module for this feature needs to be retrained. So this structure can reduce the amount of calculation and improve efficiency. The policy inference module, the action prediction module, and the state evaluation module are fully connected SNNs with two layers. Details of the two-layers SNNs are as follows. The input current of the input layer and the output layer is denoted by** ***I^in^*** **and** ***I^out^***, **respectively. The output spikes of the input layer and the output layer are denoted by** ***S^in^*** **and** ***S^out^***, **respectively. Section 3.1 describes the neural spiking process. At each time step** ***t***, **the input current to neuron** ***j*** **at the output layer is integrated as Equation (5)**.

The authors apologize for this error and state that this does not change the scientific conclusions of the article in any way. The original article has been updated.

## Publisher's Note

All claims expressed in this article are solely those of the authors and do not necessarily represent those of their affiliated organizations, or those of the publisher, the editors and the reviewers. Any product that may be evaluated in this article, or claim that may be made by its manufacturer, is not guaranteed or endorsed by the publisher.
